# Marktdominanz von Maklern auf Onlineimmobilienportalen in Deutschland und Kalifornien

**DOI:** 10.1365/s41056-022-00066-y

**Published:** 2023-01-03

**Authors:** Cedric Behler, Philip Gärtner, Hans-Joachim Linke

**Affiliations:** Technische Universität Darmstadt, Darmstadt, Deutschland

**Keywords:** Makler, Onlineimmobilienportale, Transaktionskosten, Wohnimmobilien, Immobilienmarkt, Immobilienpreise, Real estate broker, Online real estate platforms, Transaction costs, Residential real estate, Real estate market, Real estate prices

## Abstract

Der Marktanteil von Maklern als wichtige Kenngröße zum Verkaufsprozess auf dem Wohnimmobilienmarkt ist nicht erst seit der Einführung des Bestellerprinzips ein intensiv diskutiertes Thema, zu dem überraschend wenig belastbare Informationen zur Verfügung stehen. Da dieser Missstand des Informationsmangels auch bei vielen weiteren Aspekten von Immobilienmärkten auftritt, ist in jüngerer Vergangenheit verstärkt zu beobachten, dass Daten von Onlineimmobilienportalen, als am weitesten verbreiteter Vermarktungsmöglichkeit, analysiert werden. Diese Studie fügt sich mit einer vergleichenden Analyse der Maklerquote auf dem deutschen sowie dem US-amerikanischen (Teilmarkt Kalifornien) Onlineimmobilienmarkt in diese Bemühungen zur Steigerung der Transparenz ein. Hierzu wurden von marktführenden Immobilienportalen in Deutschland und den USA Angebotsdaten ausgewertet. Die statistische Analyse der insgesamt 69.711 Inserate umfasst dabei sowohl einen aggregierten Vergleich der Maklerquoten zwischen Deutschland und Kalifornien, als auch eine detaillierte Untersuchung über Unterschiede in der Häufigkeit eines Maklereinbezugs zwischen verschiedenen Marktsegmenten mittels einer logistischen Regression. Die zentrale Erkenntnis ist, dass die Maklerquote auf Immobilienportalen mit 98 % in Kalifornien signifikant über der Quote von rund 88 % in Deutschland liegt. Dies bestätigt die anhand der Analyse der Literatur abgeleitete Forschungsfrage, dass sich der Immobilienhandel in den betrachteten Ländern unterscheidet und in Kalifornien dabei häufiger Makler zum Einsatz kommen. Im Detail zeigt sich, dass in beiden Untersuchungsgebieten Eigenschaften wie Objektart und -standard einen Einfluss auf die Chance der Vermarktung mit oder ohne Makler haben. Im Ländervergleich sind dabei teils gleich- und teils gegenlaufende Effekte zu beobachten.

## Abstract (en)

The market share of brokers as an important parameter regarding the sales process in the residential real estate market has been an intensively discussed topic in Germany, on which surprisingly little reliable information is available. Since this information shortage also occurs in other aspects of real estate markets, data from online real estate platforms, as the most widespread marketing option, have been increasingly analyzed. This study complements these efforts to increase transparency with a comparative analysis of the market share of brokers in the German and US (California submarket) online real estate market. For this purpose, listing data from market-leading real estate platforms in Germany and the US were evaluated. The statistical analysis of the 69,711 listings includes both an aggregated comparison of the market share of brokers between Germany and California, as well as a detailed investigation of differences of the chance of broker involvement between different market segments using a logistic regression. The key finding is that the broker market share on real estate platforms of 98% in California is significantly higher than the rate of approximately 88% in Germany. This result confirms the research question derived from the literature analysis that real estate transactions differ in the examined countries and that brokers are used more frequently in California. In detail, it turns out that in both countries characteristics such as building type and quality have an influence on the chance of marketing with or without an agent. Comparing the two countries, these effects are partly the same and partly of an opposite nature.

## Einleitung

Kaum ein Berufszweig wird in Deutschland derart kritisch bewertet und in seiner Existenz hinterfragt wie das Maklerwesen. Regelmäßig gibt es wohnungswirtschaftliche Debatten darüber, inwiefern Makler hilfreich sind und ob ihre Vergütung als angemessen zu beurteilen ist. Auf Seiten der immobilienwirtschaftlichen Forschung existieren mannigfaltige Ansätze, Vor- bzw. Nachteile eines Immobilienmaklers im Wohnimmobiliensektor nachzuweisen, ein eindeutiges Bild zeichnet sich jedoch bis heute nicht ab (Jud und Frew 1986; Larceneux et al. 2015; Levitt und Syverson 2008; Miller et al. 2021; Stamsø 2015).

Im Spannungsfeld dieser Unsicherheit hat der deutsche Gesetzgeber auf die vorherrschenden Marktstrukturen bereits korrigierend Eingriff genommen, so etwa mit dem Ende 2020 in Kraft getretenen Gesetz über die Einführung des Bestellerprinzips bei Wohnimmobilientransaktionen, das die Verteilung der Maklerkosten reformieren sollte. Für die Beurteilung der Erforderlichkeit solcher Eingriffe sollte aus wissenschaftlicher Sicht eine fundierte Informationsgrundlage über die Relevanz von Maklern für den Transaktionsprozess Voraussetzung sein. Insbesondere der Anteil von über Makler gehandelten Immobilien scheint dafür eine geeignete Beurteilungsgrundlage zu sein. Die Quantifizierung dieses Anteils und insbesondere seine Abhängigkeit von unterschiedlichen Marktsegmenten unterliegt aber trotz der bereits von Faller et al. (2006) geäußerten Kritik auf Grund fehlender umfassender Studien weiterhin großen Unsicherheiten.

Onlineimmobilienportale, die in der Branche zu nachhaltigen Veränderungen bei der Vermarktung geführt haben (Hess und Mann 2009), können auf Grund ihrer mittlerweile sehr weiten Verbreitung herangezogen werden, um diesen Missstand zu beheben oder zumindest abzumildern. Sie liefern eine breite Datenbasis für empirische Untersuchungen, wurden jedoch bisher vergleichsweise wenig in die Forschung eingebunden (Schernthanner 2015). Erste Ansätze, die Maklerquote auf deutschen Immobilienportalen für Analysen zu verwenden, zeigen sich jedoch in der jüngeren Vergangenheit etwa bei Sagner und Voigtländer (2021) oder Toschka und Voigtländer (2017). Angebotsdaten lassen zwar keinen unmittelbaren Rückschluss auf Transaktionen zu, nähern diese jedoch an (Chapelle und Eyméoud 2022). Zudem kann über einen Vergleich mit internationalen Daten äquivalenter Herkunft eine Einordnung bzw. Validierung erfolgen, wie Bricongne et al. (2019) für Immobilienpreise zeigen. Sehr gut geeignet für eine solche Einordnung erscheint der nicht nur verhältnismäßig umfangreiche, sondern auch speziell in Bezug auf Onlineimmobilienportale gut untersuchte US-amerikanische Markt, von dem der kalifornische Markt als homogener Teilmarkt herangezogen wird.

Im Zuge dieser Studie soll daher die sich aus der breiten Datenbasis von Onlineimmobilienportalen ergebende Chance genutzt werden, um die bestehenden Unsicherheiten bezüglich des Anteils von über Makler gehandelten Immobilien zu reduzieren. Diese in jüngster Vergangenheit vermehrt in der Literatur zu beobachtende Idee, Angebotsdaten von Portalen als Marktindikatoren zu verwenden, findet sich etwa bei Bricongne et al. (2021) im Kontext von Immobilienpreisdaten. Die vorliegende Studie fügt sich damit in die Bemühungen ein, mittels Angebotsdaten zeitnahe Statistiken über den Immobilienmarkt zur Verfügung zu stellen.

Dabei ist es insbesondere von Interesse, zu prüfen, ob bestimmte Charakteristika von Immobilien einen Einfluss auf die Maklerquote haben, um festzustellen, ob es zwischen verschiedenen Marktsegmenten Differenzen in der Maklerverwendung gibt, da diese demnach einer unterschiedlichen Behandlung beispielsweise durch den Gesetzgeber bedürften. Auf Grund der hohen Unsicherheit bezüglich der Maklerquote, verursacht durch bisher nur sehr aufwändig zu erhebende Daten und in Folge fehlender wissenschaftlicher Studien, sollte der in ersten, hier bereits genannten, ähnlichen Studien verfolgte Ansatz der Auswertung von Angebotsdaten genutzt, durch einen internationalen Vergleich validiert und auf das zu Beginn beschriebene Problem der fehlenden, belastbaren Maklerquote übertragen werden. Daraus ergibt sich die zentrale Forschungsfrage dieser Studie, wie hoch die Maklerquote in Deutschland und in dem kalifornischen Vergleichsimmobilienmarkt ist und wie sie sich in unterschiedlichen Teilmärkten unterscheidet.

Im nachfolgenden Abschnitt wird zunächst auf theoretischer Ebene der deutsche und der kalifornische Immobilienmarkt, insbesondere in Bezug auf das Maklerwesen, verglichen, um daraus die Forschungsfrage, über Unterschiede in der Maklerquote allgemein und in den jeweiligen Teilmärkten im Speziellen, abzuleiten. Darüber hinaus werden Onlineimmobilienportale detailliert beleuchtet, um die Übertragbarkeit der Ergebnisse auf den Gesamtmarkt einschätzen zu können. Anschließend werden die für den empirischen Vergleich der Makleraktivität verwendeten Daten vorgestellt, die auf Onlineimmobilienportalen in Deutschland und in Kalifornien erhoben wurden. In dem darauffolgenden vierten Abschnitt wird die Methodik der Untersuchung präsentiert, anhand derer die im fünften Kapitel vorgestellten empirischen Ergebnisse gewonnen wurden. Abschließend erfolgt eine Zusammenfassung der wichtigsten Erkenntnisse.

## Makler und Immobilienportale

### Immobilientransaktionen im internationalen Vergleich

Immobilienmärkte werden gemeinhin als stark unvollkommene Märkte betrachtet (Brauer 2013), die sich international deutlich unterscheiden können. Da zur Einordnung der Analyse der Maklerquote auf dem deutschen Markt ein weiterer internationaler Markt herangezogen werden soll, müssen die Unterschiede dieser Märkte und der Maklertätigkeit im jeweiligen Markt zunächst theoretisch aufgearbeitet werden, um daraus Hypothesen zum Verhältnis der jeweiligen Maklerquoten herleiten zu können. Der US-amerikanische Markt als wissenschaftlich breit untersuchter und der kalifornische als transparenter (Jones Lang LaSalle 2018) und insbesondere regulatorisch homogener Teilmarkt^1^ sind auf Grund der genannten Transparenz und Homogenität hierfür besonders geeignet.

Dass sich die Systematik einer Transaktion in diesen beiden Ländern unterscheiden muss, zeigt sich bereits am Eigentumsnachweis. Während in den USA das Eigentum an Immobilien (*title*) über eine private Grundeigentumsurkunde (*deed*) nachgewiesen wird, deren Richtigkeit über eine Versicherung (*title insurance*) abgesichert wird, dient in Deutschland das amtlich geführte Grundbuch der lückenlosen Rückverfolgbarkeit von Grundeigentum. Die Haftung für die Richtigkeit übernimmt hierbei der Staat (Faller et al. 2006). Um die Transaktion rechtlich abzusichern und die Parteien gleichermaßen unabhängig zu beraten ist deshalb in Deutschland zwingend ein Notar zu beteiligen, der den Kaufvertrag nach den Wünschen der Parteien aufsetzt, sicherstellt, dass die Vertragsparteien die Regelungen des Kaufvertrages verstanden haben, beurkundet und eine Eintragung in das Grundbuch veranlasst. In den USA (Kalifornien) besteht hingegen keine Pflicht zur notariellen Beurkundung, jedoch ist die lückenlose Recherche der Grundstückshistorie zur sicheren Erlangung des *titles* nicht minder anspruchsvoll. Diese Aufgabe und die Erstellung des Kaufvertrags fallen daher in den USA in den Aufgabenbereich von Maklern oder Anwälten.

In den Transaktionskosten schlagen sich die Unterschiede dahingehend nieder, dass in Deutschland die Übertragung von Grundeigentum im Schnitt teurer ist. Dort fallen unumgängliche Transaktionskosten für Notar, Grunderwerbsteuer und Grundbuchgebühren an, die allein 5–8 % des Kaufpreises betragen (Voigtländer 2016). Außerdem entstehen Maklerprovisionen von üblicherweise 5–6 % netto, inklusive Umsatzsteuer bis zu 7,14 % (Toschka und Voigtländer 2017). Insgesamt kann die Belastung somit bis zu 15 % zusätzlich zum eigentlichen Kaufpreis betragen. In den USA hingegen liegen die üblichen Nebenkosten, die im Regelfall die Grunderwerbsteuer, Kosten für Anwälte und eine *title insurance* enthalten, bei 3–5 % des Kaufpreises (Global Property Guide 2020). Hinzu kommen für einen eventuell engagierten Makler weitere 6 %, was die Gesamtbelastung auf bis zu 11 % erhöht. Die gesamten Transaktionskosten sind in Deutschland demnach höher, maßgeblich getrieben durch die höhere Grunderwerbsteuer. Die Summe der Maklerkosten hingegen erscheint in beiden Ländern ähnlich. Aus einer rein rationalen Kostenperspektive betrachtet, läge daher die Vermutung nahe, dass der Arbeitsaufwand eines Maklers bei einer typischen Transaktion in den USA und Deutschland annähernd gleich sei.

Unterschiedlich gestaltet sich die Kostenübernahme. Während ein Großteil der Nebenkosten, allen voran der Makler, in den USA in der Mehrheit der Fälle durch den Verkäufer übernommen wird (National Association of Realtors 2020), ist in Deutschland das Gegenteil üblich. Zumindest für den Makler zeichnet sich in Deutschland jedoch eine Verschiebung ab, da dessen Kosten seit Ende 2020 durch das gesetzlich verankerte Bestellerprinzip nicht mehr vollständig auf den Käufer übertragen werden können, wenn der Verkäufer den Makler engagiert.

### Immobilienmakler in den USA und Deutschland

Makler vermitteln auf Immobilienmärkten zwischen dem Angebot des Verkäufers und der Nachfrage des Käufers, die aufgrund von Friktionen, insbesondere durch die Unvollkommenheit des Marktes, nicht direkt zueinander finden. Die damit verbundenen Informationsasymmetrien bewirken, dass sich Käufer und Verkäufer entweder selbst über den Markt informieren müssen oder entgeltlich einen Dritten engagieren, der dies übernimmt (Jud 1983). Diese Aufgabe können Makler dadurch bewältigen, dass sie als Experten der Branche über einen erheblichen Informationsvorsprung verfügen (Levitt and Syverson 2008).

Bei näherer Betrachtung zeigt sich im Immobiliensektor neben dem reinen Matching von Angebot und Nachfrage aber eine ganze Reihe an Funktionen des Maklerwesens, die den Aufgabenbereich in den letzten Jahrzehnten erweitert haben. Dabei entwickelt sich der Beruf immer weiter weg von der eigentlichen Vermittlerrolle hin zu einem deutlich breiter gefächerten Aufgabenspektrum, das eine ganzheitliche Beratung bei einer Immobilientransaktion beinhaltet (Henze and Knopf 2012). Gründe für die Maklerwahl sind einer empirischen Untersuchung des norwegischen Marktes von Stamsø (2015) zufolge für viele Verkäufer, dass vor allem der Aufwand des eigenständigen Verkaufs zu hoch sei, aber auch das Risiko Fehler zu machen.

Das Verhältnis zwischen Maklern und Marktteilnehmern lässt sich als Prinzipal-Agenten-Beziehung nach Jensen und Meckling (1976) beschreiben. Dabei nehmen die Käufer oder Verkäufer die Rolle des Prinzipals ein, die den Makler als Agenten beauftragen, für sie die Dienstleistung der Immobilientransaktion zu erbringen. Ein typisches Problem in dieser Beziehung entsteht, wenn Prinzipal und Agent unterschiedliche Ziele verfolgen. Dies kann bei Immobilien der Fall sein, wenn der Makler das Haus schneller verkaufen möchte, um mehr Geschäfte abschließen zu können, was in mehr Provisionen münden könnte, und deshalb einen niedrigeren Preis ansetzt als den maximal erzielbaren, den der Verkäufer anstrebt (Stamsø 2015). Relevant für die Analyse der Entscheidung, einen Makler zu engagieren, ist demzufolge die Wechselwirkung zwischen Maklern und realisierten Kaufpreisen. In der Literatur zeigt sich hinsichtlich dieses Aspektes keine Übereinstimmung der Forschungsergebnisse. Einerseits konnten einige Autoren nachweisen, dass Maklerangebote zu höheren Preisen verkauft werden als solche von privaten Verkäufern (Jud und Frew 1986; Stelk und Zumpano 2017; Voigtländer 2019; Zumpano et al. 1996). Andererseits haben Levitt und Syverson (2008) herausgefunden, dass Makler entsprechend der Prinzipal-Agenten-Theorie schneller verkaufen wollen und deshalb günstigere Preise ansetzen. Dieses Verhalten stellen auch Hendel et al. (2009) in ihrer Studie fest. Insgesamt betrachtet ist der ökonomische Einfluss des Maklerwesens nicht abschließend auszumachen. Ungeachtet dessen werden in der Praxis jedoch häufig Makler verwendet, was darauf hindeutet, dass Verkäufer und Käufer bei der Entscheidung anderen Faktoren ein höheres Gewicht beimessen. Insbesondere kommen Larceneux et al. (2015) zu dem Ergebnis, dass allein die Annahme, der Einsatz eines Maklers sei vorteilhaft, dazu führe, dass Verkäufer eher einen Makler beauftragen.

Konkret in Bezug auf die in dieser Studie betrachteten Länder zeigen sich erhebliche Differenzen im Maklerwesen. Einer der markantesten Unterschiede ist der in den USA regelmäßig anzutreffende Fall, dass sowohl der Käufer als auch der Verkäufer einen eigenen Makler engagieren, wohingegen in Deutschland üblicherweise ein Makler für beide Parteien gemeinsam tätig ist. Da durch diese Konstellation ein Makler nur die Interessen einer Partei vertritt, können sich die Kunden darauf verlassen, dass dieser sich bestmöglich für ihr Interesse einsetzt. Dagegen ist der Makler in Deutschland für beide Seiten aktiv, sodass der Kunde, teilweise berechtigt, Zweifel an der Neutralität haben kann. Das wirkt sich negativ auf den empfundenen Nutzen des Maklers aus. Außerdem bedeutet dies, dass Makler in den USA an einer Transaktion verhältnismäßig deutlich weniger verdienen als ihre deutschen Kollegen. Da die Provision für die gesamte Transaktion bei üblicherweise 6 % liegt, bekommt ein US-amerikanischer Makler effektiv rund 3 % Provision, weniger als die Hälfte der in Deutschland üblichen Provision.

Ein weiterer wesentlicher Unterschied tritt bei einem Vergleich des typischen Aufgabenspektrums hervor. Viele Aufgaben des deutschen Notars, wie die Kaufvertragserstellung und die Begleitung der Eigentumsübertragung werden in den USA mangels eines ebensolchen regelmäßig durch Makler übernommen (Faller et al. 2006). Damit versuchen die Parteien, sich eine gewisse Absicherung der Transaktion auf einem alternativen Weg einzukaufen. Die Rolle des Maklers als Experte der Branche suggeriert, dass die Transaktion über ihn sach- und fachgerecht ablaufen werde, wofür in Deutschland das Notarwesen existiert. Dadurch ist das Leistungsspektrum in den USA deutlich breiter, was ebenfalls ein besseres Preis-Leistungs-Verhältnis für den Kunden impliziert.

Einen bedeutenden Unterschied stellt weiterhin die maklerinterne Zusammenarbeit dar. US-Makler erhalten mit der Mitgliedschaft in der *National Association of Realtors (NAR) *Zugriff auf eine interne Immobiliendatenbank, *Multiple Listing Service (MLS)* genannt. Diese ist ein System aus vielen kleinen regionalen Immobiliendatenbanken für alle ansässigen Makler und enthält neben aktuellen Angeboten, die die lokalen Makler dort einstellen, auch Daten über vergangene Vertragsschlüsse, inklusive der zuletzt realisierten Kaufpreise (Hendel et al. 2009). Deutsche Makler hingegen vermitteln stets nur diejenigen Objekte, für deren Verkauf sie vorher beauftragt wurden. Insgesamt haben amerikanische Makler damit Zugriff auf ein größeres Immobilienangebot, was im Vergleich zu Deutschland ein besseres Matching ermöglicht. Zudem kann durch das *MLS* auf aktuelle Kaufpreisdaten der Umgebung zugegriffen und der Wert eines Objektes besser eingeschätzt werden. Dies steigert aus Kundensicht den Nutzen eines Maklers. Der vermutete Nutzen des *MLS* führt sogar dazu, dass in den Vereinigten Staaten einige Verkäufer den Makler einzig dazu nutzen, ihre Immobilie dort zu inserieren (National Association of Realtors 2020).

Schließlich gibt es erhebliche Unterschiede hinsichtlich der Eintrittsbarrieren in den Beruf. Diese sind in den USA bedingt durch einen verpflichtenden Lizenzierungsprozess deutlich höher als in Deutschland. Um Makler (*Broker*) zu werden, muss etwa in Kalifornien ein breites Spektrum an immobilienwirtschaftlichem, aber auch betriebswirtschaftlichem Fachwissen nachgewiesen werden (State of California, Department of Real Estate 2010). Der Berufszugang ist demnach reguliert und nicht wie in Deutschland jedermann offen, was eine grundsätzlich andere Wahrnehmung in der Öffentlichkeit auslöst. Dieses Bild zeigt sich auch in der Literatur. In Deutschland wird der Makler vorrangig als finanzielles Hemmnis für den Immobilienerwerb bewertet (Toschka und Voigtländer 2017), in den USA hingegen als Erleichterung (Hendel et al. 2009).

Die vorweg erläuterten Unterschiede deuten auf eine gänzlich verschiedene Bedeutung von Maklern für die Marktteilnehmer hin, woraus auch Unterschiede in der Häufigkeit des Maklereinbezugs abzuleiten sein könnten. Die dargestellten Argumente, die von der niedrigeren anteiligen Provision über mögliche Interessenkonflikte deutscher Makler bis hin zu den höheren Markteintrittsbarrieren amerikanischer Makler reichen, deuten alle darauf hin, dass eine Maklerbeteiligung in Kalifornien relativ gesehen vorteilhafter ist als in Deutschland und demnach üblicher sein sollte. Aus den vorgenannten theoretischen Überlegungen ergibt sich daher der erste Teil, der in der Einleitung formulierten zentralen Forschungsfrage, ob in Kalifornien Makler relativ gesehen häufiger an Immobilientransaktionen beteiligt werden als in Deutschland, da dort das Kosten-Nutzenverhältnis für den Kunden vorteilhafter erscheint.

Ein weiteres Ziel der vorliegenden Studie besteht darin, die Häufigkeit des Rückgriffs auf Makler auch zwischen verschiedenen Marktsegmenten, etwa der Vermarktung von Reihenhäusern und der Vermarktung von freistehenden Einfamilienhäusern oder der Lage im ländlichen und städtischen Raum, zu vergleichen. Stamsø (2015) und Zumpano et al. (1996) untersuchen in diesem Zusammenhang, inwiefern Charakteristika der Verkäufer wie Einkommen, Alter oder Vorerfahrung mit Transaktionen die Häufigkeit eines Maklereinbezugs beeinflussen. Daneben ist in der Literatur jedoch ein starker Fokus auf die Preis- und Zeiteffekte des Makelns zu beobachten (Benefield et al. 2014). Auf Grund dieser geringen Beachtung eines potenziellen Unterschieds von Maklerquoten in Abhängigkeit vom Marktsegment in der wissenschaftlichen Literatur und den zuvor beschriebenen, grundsätzlich für alle Marktsegmente gleichen, geltenden Rahmenbedingungen, wird der zweite Teil, der in der Einleitung formulierten zentralen Forschungsfrage, abgeleitet, ob sich die Verwendung von Maklern in den Vergleichsländern zwischen verschiedenen Marktsegmenten unterscheidet.

### Onlineimmobilienportale

Die zunehmende Verbreitung und Kommerzialisierung des Internets um die Jahrtausendwende verbesserte die Möglichkeiten des eigenständigen Verkaufs von Immobilien, die Markttransparenz erhöhte sich und es entstand ein überregionaler Marktplatz (Stamsø 2015). Maßgeblicher Treiber dieser Entwicklung waren Onlineimmobilienportale. Diese standen zunächst als neuartige Randerscheinung neben bekannten Marketingkanälen wie den Printmedien, verdrängten diese aber zusehends (Hess und Mann 2009) und erlangen daher zunehmend an Relevanz als Untersuchungsgegenstand und Informationsquelle für wissenschaftliche Auswertungen. Zur Interpretation der Ergebnisse dieser Studie sowie zur Einordnung der deutschen und der kalifornischen Ergebnisse werden die relevanten Grundlagen daher im folgenden Abschnitt beschrieben.

Wie bei der Zeitungsannonce als analogem Vorgänger ist das grundlegende Prinzip der Immobilienportale simpel. Anbieter stellen in einem ersten Schritt das zum Verkauf stehendende Objekt online in die Datenbank des Portalbetreibers ein. Suchenden wird im Folgenden auf der Internetseite des Portals entsprechend von ihnen gewählter Kriterien eine Auswahl zur Verfügung stehender Immobilien aus der Datenbank angezeigt. Der Interessent bekommt dabei zu jedem Objekt Informationen, Bilder und die Daten des Verkäufers angezeigt, sodass bei Interesse direkt Kontakt zu diesem aufgenommen werden kann. Die weiteren Schritte des Transaktionsprozesses sind schließlich Teil der persönlichen Kommunikation der beiden Parteien und erfolgen portalunabhängig.

Bereits vor der Jahrtausendwende untersuchten Buxmann und Gebauer (1998), inwiefern das Internet zu Veränderungen im Maklerwesen führen kann. Dabei wird als weiterhin plausible Haupterrungenschaft der Onlineimmobilienportale deren Fähigkeit angesehen, das einst stark lokal geprägte Geschäft mit Immobilien überregional zu öffnen. Dies zeigte sich in der Vergangenheit auch daran, dass Personen, die an einen weiter entfernten Ort umziehen und damit höhere Kosten für die Beschaffung von lokalen Marktinformationen haben, eher das Internet für die Suche nach Immobilien verwenden (Zumpano et al. 2003). Zudem steigt durch die nationale oder sogar globale Vernetzung die Reichweite von Angebot und Nachfrage. In der Folge steigt die Anzahl der Marktteilnehmer, was näher zu einem vollkommenen Markt führt. Zudem fördern die Portale als öffentlich zugängliche Datenbank an Anzeigen die Transparenz des Marktes (Schernthanner 2015). Jeder Käufer oder Verkäufer kann sich über die Preise und Eigenschaften von Immobilien in verschiedensten Regionen informieren und diese problemlos miteinander vergleichen (Enderle 2009). Dafür war in der Zeit davor stets die Expertise des Maklers notwendig.

Die Basisfunktion der *Vermittlung* ist bei internet-basierten Geschäftsmodellen wie den Onlineimmobilienportalen damit identisch zu den traditionellen Akteuren in Form von Maklern, allerdings sind die Transaktionskosten niedriger und das Matching von Angebot und Nachfrage funktioniert besser (Buxmann und Gebauer 1998). Nicht offensichtlich ist in diesem Zusammenhang, dass trotz des besseren Zusammenfindens der Marktteilnehmer die für die Suche nach dem richtigen Objekt aufgewendete Zeit durch die Verwendung des Internets nicht gesunken ist (Zumpano et al. 2003). Die von Buxmann und Gebauer (1998) vermutete Veränderung in der Rolle des Maklers mit dem Vordringen des Internets wird von Zumpano et al. (2003) bestätigt. Dabei verschiebe die Onlinesuche das Aufgabenfeld der Makler im Transaktionsprozess weiter nach hinten. Diese würden erst in einem späteren Schritt beauftragt, dann aber dabei helfen, Besichtigungen zu arrangieren und die Transaktion abzuschließen. Dabei fungiert das Internet als Vorauswahlsystem zum Finden geeigneter Immobilien, was eigentlich zum Leistungspaket eines Maklers zählt (Zumpano et al. 2003). Dieser Eindruck wird durch aktuelle Befragungen gestützt. So nutzen rund 93 % aller US-amerikanischen Immobilienkäufer im Kaufprozess Onlineimmobilienportale als Informationsquelle (National Association of Realtors 2020).

Vergleicht man die Aufgaben eines Maklers mit den Leistungen, die Immobilienportale anbieten, zeigt sich, dass letztere im Laufe der Jahre ihren Service deutlich gesteigert haben. So stellen die Portale Suchenden viele Zusatzinformationen zu einem Objekt zur Verfügung, wie etwa potenzielle Finanzierungs- oder Transaktionskosten, Standort- und Lageinformationen oder eine Einschätzung der durchschnittlichen Marktpreise in der Region. Jedoch müssen Verkäufer nach wie vor selbst aktiv werden und ihre Zeit investieren, um ein Objekt zu inserieren, wodurch Opportunitätskosten entstehen. Beauftragt man hingegen einen Makler, übernimmt dieser auf Wunsch alle Aufgaben und verspricht eine sichere Abwicklung des Geschäfts.

Aus dieser Darstellung der Unterschiede könnte der Schluss gezogen werden, dass Makler und Immobilienportale grundsätzlich als etwas Gegensätzliches zu betrachten sind. Entsprechend haben sich deutsche Makler in der Vergangenheit sehr skeptisch gegenüber der scheinbaren digitalen Konkurrenz gezeigt, da die Befürchtung eines ‚Aussterbens‘ des Maklerberufs bestand (Enderle 2009). Mit dem raschen Aufstieg der Onlineimmobilienportale wurde aber eine Nichtbeachtung und Verdrängung unmöglich. Zunehmend hat sich deshalb auch das Bild des potenziellen Konkurrenten dahingehend gewendet, dass Immobilienportale als Unterstützung und Hilfe für das eigene Geschäft angesehen werden (Enderle 2009). Hess und Mann (2009) zeigen, dass Portale bereits im Jahr 2009 mit einer Nutzungsquote von 98 % vor allen anderen Vermarktungskanälen lagen und sie damit das primäre Instrument für den Vertrieb von Immobilien für deutsche Makler darstellen.

In Kalifornien zeichnet sich ein ähnliches Bild ab. Die auch dort anfänglich vorherrschende Befürchtung der Makler, durch Onlineimmobilienportale ersetzt zu werden, hat sich, wie Zumpano et al. (2003) vermutet haben, nicht bestätigt. Dies liegt unter anderem daran, dass die Maklerbranche auf die Veränderungen reagiert hat und ihre eigene Internetpräsenz aufgebessert hat. Dazu wurde in den USA das unter Lizenz der *National Association of Realtors* betriebene Portal *realtor.com* verstärkt vermarktet und als Konkurrent zu den unabhängigen Immobilienportalen gefördert. Durch das Prinzip der gut funktionierenden lokalen *MLS-Datenbanken* bestand für amerikanische Makler lange Zeit kein unmittelbares Bedürfnis nach direktem Endkundenkontakt über das Internet. Mit der Seite *realtor.com* wurden jedoch ausgewählte Daten der lokalen *Multiple Listing Systems* auf einer gemeinsamen Webpräsenz gesammelt und an die Öffentlichkeit weitergegeben, womit ein exklusives Onlineimmobilienportal für Makler entstand (Cherif und Grant 2014). Mit diesem können alle Inserate, die die Mitglieder in ihr lokales *MLS* eingeben, online mit noch größerer Reichweite publiziert werden. In Deutschland gibt es mit dem Immobilienportal *ivd24immobilien.de* des Immobilienverbands Deutschlands eine vergleichbare, jedoch deutlich weniger bekannte Website, die ebenfalls nur Inserate von Maklern listet, die Mitglieder des Verbands sind.

Neben der Vermarktung auf berufsstandsexklusiven Portalen inserieren Makler aber auch direkt in öffentlichen Immobilienportalen. Ford et al. (2005) untersuchten ökonomische Effekte dieser zusätzlichen Vermarktung auf öffentlichen Portalen. Als Ergebnis zeigte sich, dass sich solche Objekte langsamer verkaufen als Immobilien, die nur im *MLS* gelistet waren. Diese angesichts der vergrößerten Reichweite zunächst verwunderliche Erkenntnis wird damit erklärt, dass einfach verkäufliche Objekte gar nicht erst ins Internet gestellt werden müssten. Das zeigt, dass Makler bevorzugt auf die etablierten Methoden setzen, in schwierigen Fällen jedoch gerne das Internet zu Hilfe ziehen, weshalb Ford et al. (2005) das Internet in diesem Zusammenhang treffend als „natürliche Erweiterung des MLS“ bezeichnen. In jüngster Vergangenheit sind dabei neben den bereits sehr präsenten Onlineimmobilienportalen aber auch soziale Netzwerke als Medium für die Immobilienvermarktung zu beobachten (Chiacchierini et al. 2017).

Ausgeschlossen sind Makler hingegen von speziellen *For-Sale-By-Owner(FSBO)-Portalen*. In solchen Immobilienportalen haben deshalb auch Zumpano et al. (2003) die größte Bedrohung für die Branche gesehen, jedoch ist der Erfolg dieser Websites bisher überschaubar und zumeist stark regional begrenzt. Einen konkreten Vergleich zwischen einem *MLS* und einem lokalen *FSBO-Portal* in den USA haben Hendel et al. (2009) vorgenommen. In dem dort betrachteten Zeitraum von 1998 bis 2005, also der Frühphase von Onlineimmobilienportalen, zeichnete sich bei dem prozentualen Anteil von eigenständigen Verkäufen ein positiver Trend ab (Hendel et al. 2009). Übergreifend betrachtet ist vor allem die aktuelle empirische Forschung in Bezug auf reine FSBO-Portale jedoch dünn, ein Missstand den auch Xie (2019) anmerkt.

Grundsätzlich zeigt sich aktuell, dass Käufer das Internet nutzen, um ihre Wunschimmobilie zu finden und dazu nicht mehr einen Makler beauftragen. Damit fällt dessen eigentliche Hauptaufgabe zunehmend weg (National Association of Realtors 2020). Trotzdem sind Makler im weiteren Prozess noch häufig vertreten. Auch auf der Verkäuferseite setzt sich die eigenständige Transaktion nicht mit überwiegender Mehrheit durch, was ein Indiz dafür sein könnte, dass das Zusammenbringen von Angebot und Nachfrage nicht der entscheidende Vorteil eines Maklers ist. Eigentlich sollten Transaktionen durch Immobilienportale einfacher und günstiger werden, was einen ungeheuren Vorteil des selbstständigen Verkaufs darstellen würde (Larceneux et al. 2015). Jedoch seien reine *FSBO-Portale*, vermutlich bedingt durch die geringe Netzwerkgröße, wenig effektiv hinsichtlich der Dauer bis zum Verkauf und der Wahrscheinlichkeit die Transaktion zu realisieren (Hendel et al. 2009). Der selbstständige Verkauf boome zudem auch deswegen nicht, weil Marktteilnehmer bei Maklern weiterhin Vorteile vermuten, unabhängig davon, ob diese wirklich existieren oder nicht (Larceneux et al. 2015).

Abschließend wird der für die vorliegende Studie zentrale Begriff der Maklerquote definiert. Diese sagt allgemein aus, wie viel Prozent aller Immobilientransaktionen unter Maklerbeteiligung ablaufen. Bezogen auf Onlineimmobilienportale soll die Maklerquote den Anteil der durch Makler inserierten Immobilien am gesamten Immobilienangebot des Portals bezeichnen. Das gesamte Angebot besteht dabei aus den Angeboten von Privatpersonen und denen von Maklern. Obwohl das Maklerwesen in Deutschland insbesondere in politischen Debatten regelmäßig vertreten ist, ist die empirische Forschung über die Maklerquote in Deutschland gering. An dieser bereits durch Faller et al. (2006) festgehaltenen Erkenntnis hat sich jedoch seither nicht viel verändert. Zwar schätzt der Immobilienverband Deutschland (IVD), Bundesverband der Immobilienberater, Makler, Verwalter und Sachverständigen e. V., in einer Stellungnahme zu einem Gesetzentwurf aufgrund persönlicher Erfahrungen der Mitglieder, dass rund 40 % aller Transaktionen von Wohnimmobilien von Maklern begleitet werden (Immobilienverband Deutschland 2019), diese Werte sind jedoch nicht durch eine empirische Untersuchung unterlegt. Belastbarer sind die Analysen des Gutachterausschusses für Grundstückswerte in Hamburg (2020), wonach rund 45 % aller Ein- und Zweifamilienhäuser durch Makler vermittelt werden. Die Aussagekraft dieses Werts ist jedoch auf die Großstadtsituation Hamburgs beschränkt. Schließlich wurde die Maklerquote jüngst in einem Gesetzentwurf der Bundesregierung über die Verteilung der Maklerkosten (Bundesregierung der Bundesrepublik Deutschland 2019) auf rund 43 % bei Wohnimmobilien geschätzt. Jedoch wird auch hier darauf verwiesen, dass es sich um Annahmen handelt, die nicht empirisch verifiziert wurden. Insgesamt betrachtet liegen die Schätzungen der Maklerquote auf Transaktionsebene eng beieinander, empirische Daten fehlen jedoch. Auf Angebotsebene kommunizieren Toschka und Voigtländer (2017) deutschlandweit eine Maklerquote von 59 % auf dem Immobilienportal ImmoScout24. Neueren Daten zufolge lag die Maklerquote auf Angebotsebene im Jahr 2020 bei rund 65 % (Sagner und Voigtländer 2021).

In den USA hingegen existieren empirische Untersuchungen der Maklerquote. Der eigenständige Verkauf machte laut der National Association of Realtors (2020) im Jahr 2019 nur rund 8 % aller Verkäufe aus, wohingegen 89 % über Makler verkaufen und einige Sonderfälle anderweitig, etwa an Hausankaufsgesellschaften. Ähnliche und ebenfalls empirisch ermittelte Daten liefert zudem die Forschungsabteilung eines amerikanischen Onlineimmobilienportals für das Jahr 2017. Demnach sind in der Retrospektive nur bei 11 % aller Verkäufe zu keinem Zeitpunkt des Transaktionsprozesses Makler beteiligt gewesen (Zillow 2017). Somit lässt sich für die USA vereinfachend angenommen eine Maklerquote von rund 90 % festhalten. Dies macht deutlich, dass die überwiegende Mehrheit aller Transaktionen über einen Makler verläuft und der Verkauf in Eigenregie eine unübliche Markterscheinung darstellt.

Es kann daher geschlussfolgert werden, dass Makler Onlineimmobilienportale breit nutzen und die dort inserierten Angebote keiner offensichtlichen systematischen Verzerrung zu Gunsten oder Ungunsten von Maklern unterliegen. Nichtsdestotrotz muss darauf hingewiesen werden, dass die verwendeten Daten nur eine unmittelbare Interpretation für Onlineimmobilienportale zulassen und nicht repräsentativ für den Gesamtmarkt aller gehandelten Immobilien sein müssen.

## Daten

Um die Maklerquote auf Onlineimmobilienportalen untersuchen zu können, wurden exemplarisch von jeweils marktführenden Immobilienportalen in Deutschland und in Kalifornien Informationen zu dort gelisteten Inseraten automatisiert erhoben. Eine hohe Marktabdeckung der Onlineimmobilienportale lässt sich in beiden Ländern annehmen, exakte Angaben stehen jedoch nicht zur Verfügung und Schätzungen zur Marktabdeckung führender Onlineimmobilienportale sind teils bereits mehrere Jahre alt und schwanken deutlich (Georgi und Barkow 2010; Henger und Voigtländer 2014).

Da in den USA nahezu alle Makler ein lokales *MLS* verwenden und selten direkt auf Onlineimmobilienportalen aktiv werden, greifen Portale auf die *MLS-Datenbanken* zurück und übernehmen die Inserate auf ihre Website. Einzelne *MLS* haben in der Vergangenheit die Weitergabe ihrer Daten unterdrückt, jedoch gelangen die Portale mitunter auch anderweitig an die Daten, was eine Quantifizierung dieses Problems schwierig gestaltet (Castro und Steinberg 2017). In Deutschland ergibt sich dieser Umweg nicht, da kein vergleichbares maklerinternes Kommunikationsinstrument existiert. Die Daten für den deutschen Markt umfassen Inserate aus dem gesamten Bundesgebiet, die des US-amerikanischen Marktes Inserate innerhalb des Bundesstaates Kalifornien. Sachlich erfolgte eine Beschränkung auf den Markt für Wohnimmobilien in Form von Häusern, wodurch Eigentumswohnungen ausgeschlossen wurden. Die Inserate wurden in direktem zeitlichen Zusammenhang, innerhalb eines dreimonatigen Zeitraums im Sommer/Herbst 2020, erfasst. Kombiniert konnten auf diese Weise Informationen zu 95.519 Anzeigen für Wohnimmobilien gewonnen werden, wobei 27.365 Inserate aus Deutschland und 66.154 aus Kalifornien (USA) stammen.

Als wichtigstes Merkmal im Sinne der zentralen Forschungsfrage wurde erfasst, ob ein Angebot von einem Makler oder einer Privatperson stammt. Zu den weiteren auswertungsrelevanten Merkmalen zählen Informationen über das angebotene Objekt, wie Preis, Wohnfläche, geografische Lage und Immobilientyp. Preis und Wohnfläche wurden im Datensatz in Relation gesetzt, um mittels der normierten Größe Preis pro Quadratmeter eine bessere Vergleichbarkeit von Objekten unterschiedlicher Größe zu gewährleisten. Der Immobilientyp wird im deutschen Datensatz in freistehende Einfamilienhäuser, Reihenhäuser, Doppelhaushälften und Mehrfamilienhäuser, die nicht in Wohnungseigentum unterteilt sind, unterschieden. Auf dem kalifornischen Markt finden sich mit Ausnahme von Doppelhaushälften die gleichen Kategorien. Dies begründet sich darin, dass Doppelhäuser in den USA mit weniger als 2 % aller Wohnimmobilientransaktionen eine geringe Bedeutung haben (Zillow 2017) und daher unter der Kategorie Reihenhäuser mitgelistet werden. Für die statistische Auswertung wurde die kategoriale Variable *Immobilientyp* durch mehrere Dummy-Variablen kodiert. Als Referenzkategorie wurde das freistehende Einfamilienhaus gewählt, für jede andere Kategorie wurde eine Dummy-Variable hinzugefügt, die den Wert *1* annimmt, wenn die Immobilie der Kategorie entspricht. Zuletzt wurden auf den Portalen weitere Informationen als Hilfs- und Kontrollvariablen erhoben, wozu nähere Angaben zum möglicherweise verwendeten Makler, das Datum der Anzeigenerstellung und eine portalinterne ID der Inserate gehören. Weitere Detailinformationen zu den inserierten Immobilien (Baujahr, Energieverbrauch, etc.) konnten aufgrund des Designs des Datenerhebungsprozesses nicht ermittelt werden, da aus technischen Gründen nicht jedes Inserat einzeln aufgerufen wurde, sondern lediglich Überblicksseiten mit Inseratslisten.

Zu den aus den Immobilienportalen gewonnen Daten wurde im Anschluss an die Erhebung die Variable *urban* hinzugefügt, um einen Einfluss der räumlichen Lage eines Objekts auf die Maklerquote untersuchen zu können. Dafür wurde begutachtet, ob der Kreis (Deutschland) oder das County (Kalifornien) in dem sich ein Objekt befindet, ländlich oder städtisch geprägt ist. Für diese Bewertung wurden siedlungsstrukturelle Daten des Bundesinstituts für Bau‑, Stadt- und Raumforschung (2017) für Deutschland und des U.S. Census Bureau (2010) für die USA herangezogen. Tab. 1 enthält zusammenfassend alle auswertungsrelevanten Variablen inklusive Definition der möglichen Werte im Datensatz.

**Table 1:** 

Variable	Skalierung	Definition
*makler*	Nominal (Dummy)	1, wenn Angebot von einem Makler, 0 bei Privatanbietern
*preis_pro_qm*	Metrisch	Preis pro Quadratmeter Wohnfläche in Euro
*urban*	Nominal (Dummy)	1 für Objekte im städtischen Raum, 0 im ländlichen Raum
*mfh*	Nominal (Dummy)	1, wenn Immobilie ein Mehrfamilienhaus, 0 andernfalls
*rh*	Nominal (Dummy)	1, wenn Immobilie ein Reihenhaus, 0 andernfalls
*dhh*	Nominal (Dummy)	1, wenn Immobilie eine Doppelhaushälfte, 0 andernfalls

Vor der Auswertung wurde der Datensatz bereinigt. Inserate ohne Preis oder Wohnfläche wurden ebenso entfernt wie Zwangsversteigerungsobjekte, da letztere einen fundamental verschiedenen Transaktionsprozess durchlaufen. Zudem wurden Inserate für meist nicht fertiggestellte Neubauprojekte entfernt, da diese von Bauträgern, Fertighausherstellern oder Projektentwicklern vermarktet werden. Diese sind weder als Makler im engeren Sinne noch als Privatverkäufer zu verstehen und deshalb in dieser Studie irrelevant. Weiterhin wurde eine Korrektur für das Phänomen der Inseratsweiterleitung durchgeführt. Privatpersonen, die auf weniger bekannten Immobilienportalen ihre Anzeige hochladen, können automatisch die Reichweite erhöhen, indem sie das Portal berechtigen, ihre Anzeige auf dritten Portalen ebenfalls zu inserieren. Entsprechende Anzeigen werden auf den dritten Portalen infolgedessen als scheinbare Maklerangebote angezeigt, konnten aber von uns über die Namen der Anbieter identifiziert und als privat deklariert werden. Eine Besonderheit, die ausschließlich in den USA existiert, stellt der Zustand *Pre-Foreclosure* dar. Vor einer drohenden Zwangsversteigerung aufgrund Zahlungsunfähigkeit des Kreditnehmers kann dieser selbstständig versuchen, das betroffene Objekt zu verkaufen, um den Kredit zu bedienen. Diese Angebote werden zum Teil aber nicht von den Eigentümern inseriert, sondern nach Zahlungsverzug über Bankinformationen von den Portalen automatisch generiert. Daher ist oft nicht eindeutig erkennbar, ob die Immobilien mit Hilfe eines Maklers vermarktet werden, weshalb sie auf Grund dieser Unsicherheit sowie aus Konsistenzgründen im Vergleich zu Deutschland aus der Datengrundlage bereinigt wurden. Abschließend wurden für eine bessere Vergleichbarkeit der kalifornischen Daten mit den deutschen Werten Wohnflächen von Quadratfuß in Quadratmeter und der Preis von US-Dollar in Euro umgerechnet^2^. Insgesamt enthält der, ausschließlich basierend auf den vorgenannten Gründen, bereinigte Datensatz Informationen zu 69.711 Wohnimmobilieninseraten, wovon 23.098 Angebote auf Deutschland und 46.613 auf Kalifornien entfallen, was ungefähr 84 % der Rohdaten aus Deutschland und 70 % der Rohdaten aus Kalifornien entspricht.

Tab. 2 enthält, für Deutschland und Kalifornien separat aufgeführt, die deskriptiven Statistiken der binären Variablen im Datensatz, die maßgeblich durch den Mittelwert charakterisiert werden. Da es sich um dichotome Variablen handelt, stellt dieser zugleich die relative Häufigkeit der Merkmalsausprägung *Dummy*_*i*_ *=* *1* dar und gibt damit an, wie viel Prozent aller Inserate dieses Merkmal aufweisen.

**Table 2:** 

	Deutschland (*n* = 23.098)	Kalifornien (*n* = 46.613)
Variable	Arithmetisches Mittel	Arithmetisches Mittel
*makler*	87,88 %	97,81 %
*urban*	39,90 %	94,39 %
*mfh*	20,92 %	8,72 %
*rh*	12,48 %	18,83 %
*dhh*	14,77 %	–

In der vorliegenden Stichprobe stammen rund 98 % und damit fast alle Angebote in Kalifornien von einem Makler, wohingegen es in Deutschland nur ungefähr 88 % sind. Damit liegt die Maklerquote auf US-amerikanischen Immobilienportalen rund 10 Prozentpunkte über dem deutschen Wert. Bei einem Vergleich der siedlungsstrukturellen Verteilung der Immobilien fällt auf, dass in Kalifornien rund 94 % aller Objekte dem städtischen Raum zuzuordnen sind, wohingegen es in Deutschland rund 40 % sind. Hinsichtlich des Immobilientyps zeigt sich in der Stichprobe in beiden Ländern eine überwiegende Mehrheit an freistehenden Einfamilienhäusern. Diese machen rund 73 % aller Angebote in Kalifornien und rund 52 % in Deutschland aus^3^. Die grundlegenden Verhältnisse der Wohnformen stimmen dabei mit Zensusdaten überein, was sich beispielweise an der deutlich geringeren Anzahl an Reihen‑/Doppelhäusern im Vergleich zu freistehenden Einfamilienhäusern in Kalifornien im Vergleich zu Deutschland zeigt (Statistische Ämter des Bundes und der Länder 2011; U.S. Census Bureau 2019).

Tab. 3 bildet für das einzige metrische Merkmal *preis_pro_qm* eines Angebots, zusätzlich zum arithmetischen Mittel, den Median sowie die Standardabweichung ab, um Verteilungscharakteristika beurteilen zu können.

**Table 3:** 

*preis_pro_qm* [€]	Deutschland (*n* = 23.098)	Kalifornien (*n* = 46.613)
Arithmetisches Mittel	2827,80	4217,21
Median	2466,56	3308,78
Maximum	49.166,67	85.726,12
Standardabweichung	2065,30	3554,58
Variationskoeffizient	0,73	0,84

**Figure 1:**
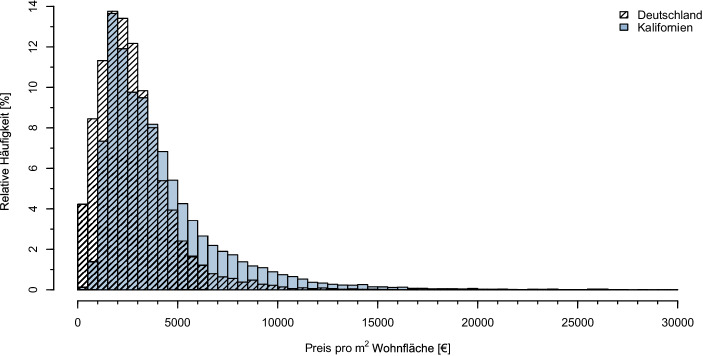

Auffällig ist der in beiden Untersuchungsgebieten auftretende deutliche Unterschied zwischen Mittelwert und Median. Dieser ist in Kalifornien stärker ausgeprägt als in Deutschland, was kohärent mit der Beobachtung von höheren Maximalwerten^4^ in Kalifornien ist. Da der Preis eine natürliche Untergrenze hat, verzerren diese den Mittelwert stärker nach oben. Bei einem Vergleich der hinsichtlich Ausreißern robusteren Mediane fällt auf, dass Wohnraum in Kalifornien für umgerechnet rund 800 €/m^2^ mehr als in Deutschland angeboten wird. Die Verteilung der Preise ist wie bereits angedeutet in beiden Untersuchungsgebieten rechtsschief, was graphisch an den flacheren rechten Seiten der Histogramme in Abb. 1 sichtbar wird. In beiden Untersuchungsgebieten liegen die Maximalwerte der relativen Verteilung in der Klasse von 2000 €/m^2^ und 2500 €/m^2^. Dass der Median in Kalifornien dennoch nicht in diese Klasse fällt, liegt maßgeblich an der breiteren Streuung der Werte in Kalifornien. Dies wird unterstrichen durch einen Vergleich der Standardabweichungen, die auf dem kalifornischen Markt ungefähr um den Faktor 1,7 größer ist als in Deutschland. Graphisch zeigt sich dies durch die im Vergleich zu den deutschen Werten weniger steil abfallende rechte Seite im Histogramm der kalifornischen Werte. Demnach liegen die Preise auf dem deutschen Angebotsmarkt enger beieinander.

## Methodik

Um zu prüfen, ob sich die Maklerquote auf Onlineimmobilienportalen in Kalifornien und Deutschland signifikant unterscheidet, wurde in einem ersten Schritt eine Kontingenzanalyse durchgeführt. Sowohl das Merkmal *Makler* als auch das Merkmal *Untersuchungsgebiet* sind nominal skaliert und haben jeweils zwei Ausprägungen. Die Variable, ob eine Immobile durch einen Makler inseriert wurde oder nicht, kann dabei die Werte *0* *=* *nein* und *1* *=* *ja* annehmen, während die Variable für das Untersuchungsgebiet zwischen *Deutschland* und *Kalifornien* wechseln kann. Durch diese Eigenschaft konnte eine Kontingenztabelle aufgestellt werden, anhand derer ein Chi-Quadrat-Test auf Unabhängigkeit (χ^2^-Unabhängigkeitstest) durchgeführt wurde.

Um in einem zweiten Schritt mögliche Auswirkungen der Eigenschaften einer Immobilie als erklärende Variablen auf die abhängige Variable der Vermarktung einer Immobilie durch einen Makler oder eine Privatperson untersuchen zu können, wurde eine logistische Regression durchgeführt. Diese spezielle Art der Regression wurde verwendet, da die abhängige Variable dichotom (binär) ist. Im Zusammenhang mit Maklerquoten findet die logistische Regression auch in verwandter immobilienwirtschaftlicher Literatur häufig Anwendung. So greifen zahlreiche Autoren, wie etwa Jud (1983), Ford et al. (2005), Stamsø (2015) und Larceneux et al. (2015), ebenfalls auf diese Form der Regression zurück, um als abhängige Variable die Entscheidung, einen Makler zu engagieren, untersuchen zu können.

Als Variablen wurden diejenigen Merkmale der Immobilien einbezogen, die von den Immobilienportalen erhoben werden konnten und bei denen ein Einfluss auf die Entscheidung des Verkäufers, für die Vermarktung einen Makler zu engagieren, vermutet wurde. Da im Rahmen der logistischen Regression die metrischen Variablen linear im Logit sein müssen (Hosmer et al. 2013), dies nach Überprüfung für die einzig metrische Variable *preis_pro_qm* aber nicht gegeben war, erfolgte eine genauere Untersuchung. Dabei zeigte sich, dass das Niveau des Quadratmeterpreises den Effekt des Quadratmeterpreises auf die abhängige Variable beeinflusst und dieser Zusammenhang annähernd quadratisch dargestellt werden kann. Demzufolge musste sowohl die Variable *preis_pro_qm* als auch das Quadrat dieser Variablen in die Regressionsfunktion aufgenommen werden.

Die zu schätzende logistische Regressionsfunktion ergibt sich mit den bereits vorweg in Tab. 1 definierten Variablen zu

1 $$\textit{makler}_{i}=\frac{1}{1+\mathrm{e}^{-\left(\beta _{0}+\beta _{1}\textit{preis}\_ \textit{pro}\_ \textit{qm}_{i}+\beta _{2}{\textit{preis}\_ \textit{pro}\_ \textit{qm}_{i}}^{2} +\beta _{3}\textit{urban}_{i}+\beta _{4}\textit{mfh}_{i}+\beta _{5}\textit{rh}_{i}+\beta _{6}\textit{dhh}_{i}\right)}} \text{f{\"u}r } i=1{,}\ldots {,}n.$$

Aufgelöst nach der systematischen Komponente entspricht dies der äquivalenten Darstellung

2 $$\ln \left(\frac{\textit{makler}_{i}}{1 - \textit{makler}_{i}}\right)=\beta _{0}+\beta _{1}\textit{preis}\_ \textit{pro}\_ \textit{qm}_{i}+\beta _{2}{\textit{preis}\_ \textit{pro}\_ \textit{qm}_{i}}^{2}+\beta _{3}\textit{urban}_{i}+\beta _{4}\textit{mfh}_{i}+\beta _{5}\textit{rh}_{i}+\beta _{6}\textit{dhh}_{i}\ \textit{f{\"u}r}\ i=1{,}\ldots {,}n.$$

Im Modell wird mit der abhängigen Variable *makler*_*i*_ die Erfolgswahrscheinlichkeit bezeichnet, dass ein Inserat einer Immobilie mit bestimmten Eigenschaften von einem Makler stammt. Diese kann im Datensatz nur die Werte *0* und *1* annehmen, weil lediglich das Ergebnis, also ob ein Inserat von einem Makler angeboten wird oder nicht, beobachtet werden kann, nicht jedoch die Wahrscheinlichkeit des einzelnen Inserats ex ante.

Die geschätzten Koeffizienten *β*_*j*_ einer logistischen Regression entziehen sich einer direkten und einfachen Interpretation durch die Logit-Transformation. Bei einer Veränderung einer unabhängigen Variablen *x*_*j*_ um eine Einheit, verändert sich der Logit, also der natürliche Logarithmus der Chance^5^, um *β*_*j*_ Einheiten. Für eine bessere Interpretierbarkeit dieser Werte erfolgt, abhängig vom Skalenniveau der einzelnen unabhängigen Variablen, eine Transformation der geschätzten Parameter. Für dichotome erklärende Variablen empfehlen Hosmer et al. (2013) eine Betrachtung der ebenfalls in FN 5 definierten Odds-Ratio (Chancenverhältnis), die in diesem Fall

3 $$\mathrm{OR}=\ \mathrm{e}^{{\beta _{j}}}\quad \mathrm{bzw}.\quad \widehat{\mathrm{OR}}=\ \mathrm{e}^{\widehat{\beta _{j}}}$$

beträgt. Das Chancenverhältnis kann dann direkt interpretiert werden als derjenige Faktor, um den die Chance (Odds) steigt, wenn die dichotome Variable statt *x*_*j*_ = 0 den Wert *x*_*j*_ = 1 annimmt (Backhaus et al. 2018). Dabei darf die Odds-Ratio nicht mit dem Faktor, um den die Wahrscheinlichkeit steigt, verwechselt werden, d. h. Odds und Wahrscheinlichkeiten sind stets strikt zu trennen. Bei kategorialen unabhängigen Variablen, die durch mehrere dichotome Variablen kodiert werden, ist die Interpretation ähnlich. Dabei wird die Odds-Ratio jeweils als Steigerung der Odds bei einem Wechsel von der Referenzkategorie zu der durch die Dummy-Variablen kodierten Kategorie betrachtet.

Schließlich gilt es den Fall metrischer erklärender Variablen zu betrachten. Bei diesen hat die Einheit bzw. Größenordnung der Variablen einen erheblichen Einfluss auf die Interpretation. Theoretisch kann erneut das Chancenverhältnis aufgestellt werden, dieses beziffert jedoch nur die Veränderung bei einer Erhöhung um eine Einheit. Bewegt sich die Variable *x*_*j*_ jedoch im Rahmen von Hunderttausendern, ist eine Erhöhung um eine Einheit keine bedeutende Veränderung. Deshalb gilt es, eine Konstante c derart zu wählen, dass eine Erhöhung um *c* Einheiten relevant ist. Dann trifft die Odds-Ratio

4 $$\widehat{\mathrm{OR}}\left(\mathrm{c}\right)=\ \mathrm{e}^{\mathrm{c}\widehat{\beta _{j}}}$$

eine Aussage über eine Erhöhung von *x*_*j*_ um *c* Einheiten (Hosmer et al. 2013).

## Empirische Ergebnisse

### Analyse der Maklerquote auf Ebene der Untersuchungsgebiete

Die in Tab. 4 dargestellte Kontingenztafel beschreibt die Maklerquote auf Onlineimmobilienportalen in Kalifornien und Deutschland. Zeilensummen und -prozente kennzeichnen die Anteile der durch Makler angebotenen Objekte in jedem Untersuchungsgebiet. Die Stichprobengröße von fast 70.000 Inseraten erfüllt die Voraussetzungen für eine uneingeschränkte Anwendung des approximativen χ^2^-Unabhängigkeitstests. Dabei wird auf einem Signifikanzniveau von α = 1 % geprüft, ob die Nullhypothese des hier formulierten χ^2^-Unabhängigkeitstests, dass die Merkmale *Untersuchungsgebiet des Verkaufs* und *Vermarktung der Immobilie durch Makler* unabhängig sind, verworfen werden kann.

**Table 4:** 

		Immobilie inseriert durch Makler		
		Nein (0)	Ja (1)	Zeilensumme
Untersuchungsgebiet	Deutschland	2799 (12,12 %)	20.299 (87,88 %)	23.098 (100 %)
	Kalifornien	1023 (2,20 %)	45.590 (97,81 %)	46.613 (100 %)
	Spaltensumme	3822	65.889	69.711

Anmerkungen. Werte in Klammern entsprechen relativen Häufigkeiten in Zeilenprozenten

Aus der Teststatistik *T* des χ^2^-Unabhängigkeitstests sowie dem kritischen Wert ${\chi }_{1-\upalpha}^{2}\left(5\right)$ folgt, dass die Nullhypothese, dass Inserate auf Onlineimmobilienportalen in Kalifornien und Deutschland gleich häufig von Maklern stammen, verworfen werden kann:

5 $$\mathrm{T}\approx 2.889{,}505> 6{,}635={\chi }_{0{,}99}^{2}$$

Folglich ist die kalifornische Maklerquote auf Immobilienportalen mit einer Irrtumswahrscheinlichkeit von 1 % von der deutschen statistisch signifikant verschieden. Die Richtung des Unterschieds lässt sich anhand der Zeilenprozente in Tab. 4 ablesen, wonach der Anteil der durch Makler angebotenen Objekte auf Immobilienportalen in Kalifornien größer ist als in Deutschland. Dies beantwortet den in Abschn. 2.2 anhand der ermittelten Unterschiede im Maklerwesen aufgestellten ersten Teil der zentralen Forschungsfrage.

Die beobachteten Anteile zeigen auch, dass auf Immobilienportalen auf Angebotsebene die Maklerquote höher ist als auf dem Transaktionsmarkt. So steht in Kalifornien den in dieser Studie beobachteten 98 % maklerbetreuten Angeboten auf Immobilienportalen eine in Abschn. 2.3 anhand von vorhandener Literatur geschätzten Maklerquote von rund 90 % aller Transaktionen gegenüber. Äquivalent betragen die Werte für den deutschen Raum rund 88 % auf Portalen und zwischen 40 und 45 % in der Literatur. Ansätze zur Erklärung dieser Unterschiede könnten sein, dass zwar die meisten Angebote von Maklern auf Portalen gelistet werden, Verkäufer, die direkt an Bekannte und Verwandte veräußern, ihr Haus jedoch gar nicht erst inserieren. Darüber hinaus könnten andere Schätzungen auf anderen Daten basieren und beispielsweise Projektentwicklungen oder Zwangsversteigerungen als nicht durch Makler veräußerte Objekte enthalten, die in diesem Fall bewusst bereinigt wurden. Auf Grund der in beiden Untersuchungsgebieten durchgeführten Bereinigung mit Ausschluss von mindestens 15 % der Daten, könnte dies einen relevanten Einfluss auf die Analyse gehabt haben. In Kalifornien ist der Anteil der so verkauften Immobilien bei einem Vergleich der Literatur mit den empirischen Ergebnissen recht gering, in Deutschland hingegen gibt es enorme Differenzen. Fraglich ist, ob dieser Effekt in Deutschland wirklich bedeutend größer ist und mehr Personen mittels alternativer Kanäle verkaufen, oder ob die Schätzungen der Maklerquote auf dem deutschen Markt unzutreffend sein könnten. Ein möglicher Grund dafür, dass die Differenz in Deutschland größer ist als in Kalifornien, könnte an der benötigten Unterstützung beim Verkauf liegen. Verkäufer in Kalifornien engagieren Makler oft auch, weil es keinen Notar gibt und sie Hilfe bei der Abwicklung der Transaktion benötigen. In Deutschland ist durch die Stelle des Notars die Leistung des Maklers eher auf die Vermarktung bezogen. Dieser Grund fällt z. B. bei Verkäufen unter Freunden weg, da man die andere Vertragspartei kennt, wohingegen in Kalifornien die Unsicherheit bei den Spezifika des Transaktionsprozesses weiter fortbesteht. Daraus folgt, dass in Kalifornien zwar möglicherweise ähnlich häufig wie in Deutschland an Freunde und Verwandte verkauft wird, dabei jedoch manchmal dennoch ein Makler eingeschaltet wird.

Es ist aber auch möglich, dass die geschätzten Werte in der Literatur zu niedrig angesetzt sind. Dies hätte direkte Auswirkungen auf die aktuelle politische Debatte über die Einführung des Bestellerprinzips bei Verkäufen von Wohnimmobilien, das wie bereits erwähnt Mitte 2020 beschlossen wurde. Ein Anliegen der Einführung war, die Käufer vor der Zwangslage zu bewahren, keine Angebote ohne Makler mehr zu finden (Bundesregierung der Bundesrepublik Deutschland 2019). Im Zuge dessen hat der IVD als Stimme der Makler in einer Stellungnahme auf Grund einer eigens geschätzten Maklerquote von 40 % diese Zwangslage entschieden verneint (Immobilienverband Deutschland 2019). Ergebnisunabhängig zeigt sich die Relevanz belastbarer Aussagen zur Maklerquote.

Ein Vergleich der aktuellen Maklerquote mit historischen Werten, ist in Deutschland bedauerlicherweise nicht möglich, da für die Vergangenheit keine Daten existieren. In den USA hingegen wird die Maklerquote schon seit den 1980iger Jahren von der *National Association of Realtors* statistisch ausgewertet. So beziehen sich Jud und Frew (1986) auf diese Daten aus dem Jahr 1981, die den aktuellen Zahlen in der Erhebungsart entsprechen und somit gut vergleichbar sind. Damals haben rund 85 % aller Transaktionen unter Maklerbeteiligung stattgefunden. In den neunziger Jahren, also vor dem großen Internetboom und dem Aufkommen der Onlineimmobilienportale, lagen die Maklerquoten in den USA Daten der National Association of Realtors (2014) zufolge bei rund 80 %. Zu beobachten ist seitdem ein kontinuierlicher Abfall der FSBO-Geschäfte von ehemals 19 % im Jahr 1991 und damit ein stetiger Anstieg der Maklerquote. Dies ist insbesondere vor dem Kontext, dass mit dem Aufstieg der Immobilienportale als neue, reichweitenstarke Vermarktungsmöglichkeit für den Privatverkäufer der eigenständige Verkauf eher einfacher geworden ist, verwunderlich.

### Einflussfaktoren der Entscheidung über einen Maklereinbezug

Die Ergebnisse der logistischen Regression sind für den deutschen Datensatz in Tab. 5 und für Kalifornien in Tab. 6 dargestellt. In den Tabellen sind in der zweiten Spalte die geschätzten Koeffizienten dargestellt, die in dieser Form jedoch schlecht zu interpretieren sind. Daher erfolgt eine Transformation und Auswertung der einzelnen Koeffizienten ausführlicher in den nachfolgenden Abschnitten. Die Teststatistiken des Wald-Tests in der vierten Spalte sowie die zugehörigen *p*-Werte sind dabei wie bei einem gewöhnlichen *t*-Test im Rahmen der linearen Regression zu interpretieren. Geringe *p*-Werte implizieren, dass die Koeffizientenschätzwerte signifikant von Null verschieden sind.

**Table 5:** 

Deutschland (*n* = 23.098)					
unabhängige Variable	Koeffizient	Standard- abweichung	Wald- Teststatistik	*p*-Wert	
	$\upbeta$		*z*	Pr(> |*z*|)	
*(Konstante)*	2,4448	0,0739	33,095	< 0,0001	**
*preis_pro_qm*	−0,0003	< 0,0001	−8,177	< 0,0001	**
*preis_pro_qm^2*	2,3e‑8	< 0,0001	6,656	< 0,0001	**
*urban*	0,0984	0,0450	2,188	0,0287	*
*mfh*	0,2584	0,0589	4,388	< 0,0001	**
*rh*	−0,2916	0,0584	−4,995	< 0,0001	**
*dhh*	−0,0517	0,0584	−0,885	0,3762	–

* Signifikant auf dem 5 %-Niveau, ** signifikant auf dem 1 %-Niveau

Referenzkategorie: freistehendes Einfamilienhaus im ländlichen Raum

**Table 6:** 

Kalifornien (*n* = 46.613)					
unabhängige Variable	Koeffizient	Standard- abweichung	Wald- Teststatistik	*p*-Wert	
	$\upbeta$		*z*	Pr(> |*z*|)	
*(Konstante)*	2,8038	0,1037	27,026	< 0,0001	**
*preis_pro_qm*	0,0002	< 0,0001	−8,177	< 0,0001	**
*preis_pro_qm^2*	−2,3e‑9	< 0,0001	6,656	< 0,0001	**
*urban*	0,3498	0,1066	2,188	0,0010	**
*mfh*	0,3455	0,1268	4,388	0,0064	**
*rh*	0,7740	0,1136	−0,885	< 0,0001	**

* Signifikant auf dem 5 %-Niveau, ** signifikant auf dem 1 %-Niveau

Referenzkategorie: freistehendes Einfamilienhaus im ländlichen Raum; Variable *dhh* in Variable *rh* enthalten

Die Güte der Regression wird, wie von Backhaus et al. (2018) für die logistische Regression empfohlen, über den Likelihood-Ratio-Test geprüft. Dieser gibt für beide Regressionsmodelle hohe Teststatistiken und demnach geringe *p*-Werte von deutlich weniger als 0,1 % aus. Damit kann für beide Länder die Nullhypothese, dass das jeweilige Modell keine Erklärungskraft besitze, auf einem Signifikanzniveau von 1 % verworfen werden und der Erklärungsgehalt der Modelle als hoch signifikant eingeordnet werden.

#### Einfluss des Immobilienpreises

Der Preis je Quadratmeter Wohnfläche einer Immobilie hängt nichtlinear mit dem Logit zusammen. In der Folge wurde sowohl die Variable als auch deren Quadrat in die Regression aufgenommen. Um den Effekt des Preises auf die abhängige Variable zu interpretieren, wird die vorweg erläuterte Odds-Ratio berechnet. Diese ist für eine bessere Interpretierbarkeit für metrische Variablen für eine Veränderung von *c* Einheiten zu betrachten. Da mehrere Koeffizienten gleichzeitig interpretiert werden müssen, kann die in Abschn. 4 verwendete, einfache Formel nicht angesetzt werden. Stattdessen wird die modifizierte Formel

6 $$\widehat{\mathrm{OR}}\left(c\right)=\mathrm{e}^{{c\hat{\beta }_{1}}+c{\hat{\beta }_{2}}\left(2x_{1}+c\right)}$$

genutzt, deren Herleitung im Anhang angegeben ist. Dabei wird ein Wert von *c* = 100 €/m^2^ als Interpretationsgrundlage für die Veränderung des Preises pro Quadratmeter gewählt. Einsetzen von *x*_*1*_ *=* *preis_pro_qm*_*i*_ und den geschätzten Koeffizienten^6^ ergibt für Deutschland

7 $$\widehat{\mathrm{OR}}_{\mathrm{D}}\left(c=100\right)=\mathrm{e}^{100\mathrm{*}(\hbox{--}0{,}000265449)+100\mathrm{*}0.000000023\mathrm{*}(2\mathrm{*}{\textit{preis}\_ \textit{pro}\_ \textit{qm}_{i}}+100)}$$

und für Kalifornien

8 $$\widehat{\mathrm{OR}}_{\mathrm{K}}\left(c=100\right)=\mathrm{e}^{100\mathrm{*}0.000161775+100\mathrm{*}(\hbox{--}0.000000002)\mathrm{*}(2\mathrm{*}{\textit{preis}\_ \textit{pro}\_ \textit{qm}_{i}}+100)}.$$

**Figure 2:**
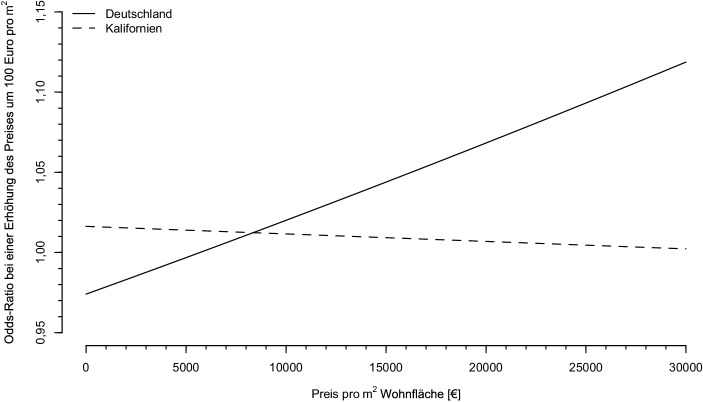

Die Odds-Ratio ist demnach eine Funktion der Variable *preis_pro_qm*_*i*_ und lässt sich als solche in einem Koordinatensystem abbilden, wie in Abb. 2 dargestellt. Die Kurve der kalifornischen Daten zeigt, dass der Preis pro Quadratmeter und damit die Wertigkeit einer Immobilie in Kalifornien nur einen sehr geringen Einfluss auf die Chance hat, dass ein Angebot von einem Makler inseriert wurde. Da die Kurve nahe an 1 liegt und die Odds mit diesem Faktor multipliziert werden, bedeutet dies, dass die Chancen bei unterschiedlichen Preisen fast unverändert bleiben. Die Odds-Ratio sagt in diesem Zusammenhang jedoch entsprechend der Erläuterungen in Abschn. 4 nichts zum Verhältnis der Wahrscheinlichkeiten aus, sie lässt also keine Rückschlüsse zum Verhältnis der Wahrscheinlichkeit einer Maklerbeteiligung zu. Der schwache negative Trend deutet an, dass bei niedrigen und mittleren Preisniveaus steigende Preise die Chancen auf eine Maklerbeteiligung stärker erhöhen als bei hohen Preisen. Im Spitzenpreisniveau nehmen die Chancen durch eine weitere Preiserhöhung dann sogar ab. Somit ergibt sich ein Maximum der Maklernutzung kurz unterhalb des teuersten Preissegments.

Für Deutschland hingegen weist die Kurve einen positiven Trend auf. Bei einem geringen Preisniveau wirkt sich eine Steigerung um 100 €/m^2^ chancenvermindernd aus, dieser Trend schlägt jedoch um, sodass im weiteren Verlauf bei höheren Preisniveaus eine Erhöhung um 100 €/m^2^ die Odds, dass ein Inserat von einem Makler stammt, um mehr als das 1,1-fache erhöht. Dies bedeutet, dass in Deutschland sowohl extrem günstige als auch sehr hochpreisige Häuser häufiger von Maklern angeboten werden und es dazwischen, dort wo die Odds-Ratio gleich 1 ist, ein Minimum gibt.

Dies kann derart interpretiert werden, dass Objekte mit sehr niedrigem oder sehr hohem Preis am schwierigsten zu vermarkten sind, da sie z. B. für Käufer durch anstehende Renovierungen wenig attraktiv sind (sehr günstige Inserate) oder der Kreis der Nachfrager sehr klein ist (sehr teure Inserate). In Kalifornien scheint genau das Gegenteil der Fall zu sein, da dort Objekte aus dem mittleren bis gehobenen Preisniveau am häufigsten über Makler angeboten wurden, während bei Höchstpreisen wieder vermehrt selbst verkauft wird. Dieser Trend ist jedoch recht schwach und könnte sich dadurch erklären lassen, dass entsprechend Stamsø (2015) und Zumpano et al. (1996) für steigende Haushaltseinkommen, was in ihren Studien als Proxy für höherwertige Immobilien betrachtet werden kann, die Maklerbeteiligung ansteigt. Schwer erklärbar in den Daten ist, warum es nach den Ergebnissen der logistischen Regression bei extrem teuren Immobilien in Kalifornien unwahrscheinlicher wird, dass diese per Makler verkauft werden. Eine mögliche Erklärung könnte sein, dass bei sehr hohen Preisen die gleiche prozentuale Provision zu höheren absoluten Provisionen bei jedoch gleicher Leistung des Maklers führt und ein Missverhältnis zwischen Preis und Leistung wahrgenommen wird. Zudem sind Personen, die hochpreisige Immobilien verkaufen, im Durchschnitt gebildeter (Young 2017), weshalb eventuell weniger Unterstützung benötigt wird.

Vergleicht man den Einfluss eines Maklers auf den durchschnittlichen Angebotspreis pro Quadratmeter Wohnfläche eines Inserats, zeigt sich auch hier in beiden Ländern ein gegensätzlicher Einfluss. Tab. 7 lässt sich entnehmen, dass maklerbetreute Inserate in Deutschland mit einem im Schnitt rund 250 €/m^2^ niedrigeren Preis inseriert sind als private Angebote und dieser Unterschied hoch signifikant ist. Dies könnte einerseits wiederum mit dem bereits vorgebrachten Erklärungsansatz in Verbindung gebracht werden, dass günstige Inserate (z. B. mit Instandhaltungsrückstau) möglicherweise schwerer zu vermarkten sind und deswegen häufiger die professionelle Hilfe eines Maklers in Anspruch genommen wird. Andererseits bestätigt das Verhalten in Deutschland auch die Ergebnisse von Levitt und Syverson (2008), die in günstigeren Maklerangeboten den Versuch der Makler erkennen, eine Transaktion schneller herbeizuführen.

**Table 7:** 

		Immobilie inseriert durch Makler		Welch-Teststatistik	*p*-Wert	
		Nein (0)	Ja (1)	*t*	Pr(> |*t*|)	
Untersuchungsgebiet	Deutschland	3047,56 €/m^2^	2797,49 €/m^2^	6,86	< 0,0001	**
	Kalifornien	3240,38 €/m^2^	4239,13 €/m^2^	−10,06	< 0,0001	**

** Der Welch-Test auf Gleichheit der Mittelwerte in zwei Stichproben ist signifikant auf dem 1 %-Niveau

In Kalifornien ist im Gegensatz dazu zu beobachten, dass Inserate, die von Maklern stammen, im Schnitt umgerechnet rund 1000 €/m^2^ teurer sind als Inserate von Privatpersonen. Dieses ebenfalls hoch signifikante Ergebnis lässt sich konsistent mit den vorigen Ergebnissen dieses Abschnitts derart interpretieren, dass es in der (gehobenen) Mittelschicht in Kalifornien üblich sein könnte, einen Makler zu engagieren und Marktteilnehmer, die dies nicht tun, negativ auffallen. Die Reservationspreise der Käufer für derartige Angebote könnten aufgrund erhöhter Risiken bzw. Unsicherheiten bei der Transaktion niedriger ausfallen. Dementsprechend müssten private Verkäufer ihre Immobilien günstiger anbieten, um sie zu verkaufen. Die Erkenntnis, dass in Kalifornien höhere Preise bei maklerbetreuten Inseraten anfallen, ist zudem konsistent mit den Erkenntnissen von Jud and Frew (1986) oder Stelk und Zumpano (2017).

Abschließend lässt sich in Bezug auf die Preisvariable im Verhältnis zu einer potenziellen Maklerbeteiligung festhalten, dass sich, wie bereits in Abschn. 2.2 angedeutet, in den Ergebnissen in der einschlägigen Literatur kein konsistentes Verhalten feststellen lässt. Zu dieser Erkenntnis gelangen auch Benefield et al. (2014), die dies insbesondere auf die Vielzahl an angewendeten Methoden zurückführen.

#### Einfluss des siedlungsstrukturellen Raumtyps

Der Einfluss der Lage einer Immobilie im städtischen oder ländlichen Raum ist sowohl in Deutschland als auch in Kalifornien mindestens auf dem 5 %-Niveau signifikant, wie sich an den *p*-Werten von 0,0287 und 0,001 zeigt. Durch eine Transformation der Koeffizienten kann der Effekt erneut als Odds-Ratio interpretiert werden. Die Werte ergeben sich zu

9 $$\widehat{\mathrm{OR}}_{\text{Deutschland}\,}=\mathrm{e}^{0{,}0984}\approx 1{,}103\ \mathrm{und}\ \widehat{\mathrm{OR}}_{\text{Kalifornien}\,}=\mathrm{e}^{0{,}3498}\approx 1{,}419.$$

Da die Referenzkategorie (*urban* = 0) den ländlichen Raum repräsentiert und die Chancenverhältnisse jeweils > 1 sind, hat ein Eintreten des Ereignisses *urban* = 1 in beiden Ländern einen positiven Einfluss auf die Odds, dass ein Inserat von einem Makler stammt. Liegt ein Objekt demnach im städtischen Raum, steigert dies die Chance, dass es von einem Makler inseriert wurde, in Deutschland ungefähr um den Faktor 1,1 und in Kalifornien um 1,4 gegenüber der Chance, die besteht, wenn es sich um ein Objekt aus dem ländlichen Raum handelt. Sowohl in Kalifornien als auch in Deutschland stammen somit Inserate in städtischer Lage häufiger von Maklern als auf dem Land. Zu ähnlichen Ergebnissen kommen auch die Untersuchungen von Stamsø (2015) und Zillow (2017), wohingegen Larceneux et al. (2015) keinen Einfluss der Lage ausmachen können. Insgesamt betrachtet ist der Effekt der siedlungstrukturellen Lage eines Objektes in Kalifornien zudem statistisch signifikant größer als in Deutschland^7^.

#### Einfluss des Immobilientyps

Die Referenzkategorie bei der Analyse der Wohnimmobilientypen in dieser Studie stellt das freistehende Einfamilienhaus dar, welches in beiden Datensätzen, gemessen an der Zahl der Inserate, die am häufigsten vertretene Kategorie ausmacht. Durch die Kodierung über Dummy-Variablen zeigen die Ergebnisse, wie sich die Maklerquote verändert, wenn statt einem freistehenden Einfamilienhaus ein anderer Typ inseriert wird. In Kalifornien sind die Koeffizienten für Reihenhäuser und Mehrfamilienhäuser statistisch hoch signifikant, wie sich an den *p*-Werten von weniger als 1 % in Tab. 6 zeigt. In Deutschland zeigt sich ein ähnliches Bild. Auch hier sind die Koeffizienten für Reihen- und Mehrfamilienhäuser durch niedrige *p*-Werte in Tab. 5 gekennzeichnet und damit wiederum hoch signifikant. Lediglich der Koeffizient für Doppelhaushälften ist mit einem *p*-Wert von 0,3762 nicht signifikant. Folglich scheint es keinen Unterschied zwischen freistehenden Einfamilienhäusern und Doppelhaushälften zu geben, was die Maklerquote bei Inseraten angeht. Um die restlichen Koeffizienten besser interpretieren zu können, erfolgt erneut eine Transformation in Odds-Ratios, wie in Tab. 8 dargestellt.

**Table 8:** 

Variable	Deutschland		Kalifornien		Welch-Teststatistik	*p*-Wert	
	$\hat{\upbeta }$	$\mathrm{e}^{\hat{\upbeta }}=\widehat{\mathrm{OR}}$	$\hat{\upbeta }$	$\mathrm{e}^{\hat{\upbeta }}=\widehat{\mathrm{OR}}$	*t*	Pr(> |*t*|)	
*mfh*	0,2584	1,2948	0,3455	1,4127	−123,85	< 0,0001	**
*rh*	−0,2916	0,7471	0,7740	2,1684	−1635,6	< 0,0001	**

Anmerkungen. Welch-Test auf Gleichheit der Koeffizienten mittels deren geschätzter Standardabweichungen

** Signifikant auf dem 1 %-Niveau

Es zeigt sich, dass die Chancenverhältnisse ($\widehat{\mathrm{OR}}$) in Kalifornien und Deutschland für Mehrfamilienhäuser beide über der Schwelle von 1 liegen. Somit sind in beiden Ländern die Chancen höher, dass eine Immobilie von einem Makler angeboten wird, wenn ein Objekt ein Mehrfamilienhaus statt ein Einfamilienhaus ist. Dies könnte darauf zurückzuführen sein, dass Mehrfamilienhäuser einen kleineren Interessentenkreis haben und damit schwerer zu vermarkten sind oder eher als Investitionsobjekte gesehen werden, bei denen eine Maklerbeteiligung branchenüblich sein könnte. Dabei ist der Effekt in Kalifornien statistisch signifikant größer.

Entgegengesetzte Erkenntnisse treten bei Reihenhäusern auf. In Deutschland vermindert sich bei diesen die Chance, dass das Objekt von einem Makler inseriert wurde, ungefähr mit dem Faktor 0,75 (ist also um ungefähr 0,25 geringer) im Vergleich zu freistehenden Einfamilienhäusern. In Kalifornien hingegen ist genau das Gegenteil zu beobachten, da dort die Chance bei Reihenhäusern mehr als doppelt so groß ist. Die Koeffizienten unterscheiden sich statistisch hoch signifikant und bestätigen somit den gegenläufigen Einfluss der Variable *rh* in Deutschland und Kalifornien. Demnach werden Reihenhäuser in Kalifornien auf Immobilienportalen deutlich häufiger von Maklern angeboten als freistehende Einfamilienhäuser, während in Deutschland der umgekehrte Fall zu beobachten ist. Dabei ist zu beachten, dass in Kalifornien in der Kategorie der Reihenhäuser auch vereinzelt Doppelhaushälften zu finden sind, deren Effekt wie bereits erwähnt nicht separat ermittelt werden konnte. Ein möglicher Grund für diese gegensätzliche Beobachtung könnte sein, dass Reihenhäuser in den USA deutlich seltener und unbeliebter sind als in Deutschland. In einer Befragung amerikanischer Hauskäufer äußerten etwa 84 % aller Käufer Interesse an einem freistehenden Einfamilienhaus, wohingegen Eigentumswohnungen, Reihenhäuser und Doppelhäuser nur weniger als jeder achte erstrebenswert findet (Zillow 2017). Reihenhäuser sind in den USA demnach schwerer zu vermarkten, weshalb eher Hilfe hinzugezogen wird. In Deutschland hingegen ist das Reihenhaus insbesondere auch bei preisbewussten Personen beliebt. Diese beauftragen daher womöglich eher seltener einen Makler, um die fällige Provision einzusparen.

Der zweite Teil der zentralen Forschungsfrage, dass die Maklerquote vom Marktsegment unabhängig ist, muss daher verneint werden, da sowohl Preis, siedlungsstruktureller Raumtyp als auch Immobilientyp einen Einfluss auf die Chance der Verwendung eines Maklers haben. Der Einfluss unterscheidet sich zwischen den Vergleichsländern und validiert sich somit nicht, weshalb in ergänzenden Studien zu überprüfen ist, ob er reproduzierbar ist und als belastbarer, sich unterscheidender Zusammenhang in den jeweiligen Ländern angesehen werden kann.

## Fazit

Die im Zuge der Verbreitung des Internets entwickelten Onlineimmobilienportale haben die Strukturen des traditionell lokalen Immobilienmarkts aufgebrochen und für mehr Transparenz gesorgt. Die anfängliche Befürchtung der Makler, ersetzt zu werden, hat sich dabei nicht bestätigt, vielmehr sind Onlineimmobilienportale mittlerweile zu ihrem wichtigsten Instrument in der Vermarktung geworden. Wie stark Immobilienportale heute durch Makler geprägt sind und wie viel Raum dabei für private Verkäufer verblieben ist, wurde in der vorliegenden Arbeit exemplarisch für Kalifornien und Deutschland untersucht. Die dabei von führenden Immobilienportalen in den USA und Deutschland erhobenen Datensätze zeigen in Bezug auf die Beantwortung des ersten Teils der Forschungsfrage, dass mit einer Maklerquote von 98 % in Kalifornien deutlich mehr Objekte durch Makler vermarktet werden als in Deutschland, wo die Maklerquote 88 % beträgt. Insgesamt betrachtet wird damit ein enormer Anteil der Verkaufsanzeigen auf Onlineimmobilienportalen von Maklern geschaltet. Dieser Eindruck wird dadurch verstärkt, dass Verkäufer, die ihre Immobilie über andere Kanäle verkaufen, das Objekt nicht ins Internet stellen. Kaufinteressenten mit unzureichenden Beziehungen fällt es daher sehr schwer, nicht durch Makler betreute Immobilien zu finden. Der große Vorteil der Immobilienportale, das ehemals lokale Geschäft mit Immobilien überregional zu öffnen, kann sich bei solchen Geschäften ohne Makler nur unzureichend entfalten, da diese zu einem großen Teil gar nicht erst ins Internet gelangen.

Bei der Analyse der Einflussfaktoren (*Preis pro Quadratmeter Wohnfläche, räumliche Lage *und* Immobilientyp*) auf die Maklerquote innerhalb der Länder, hat sich gezeigt, dass alle betrachteten Eigenschaften einer Immobilie einen signifikanten Einfluss auf die Chance haben, dass ein Objekt von einem Makler inseriert wird. In Bezug auf den zweiten Teil der Forschungsfrage lässt sich daher feststellen, dass die Maklerbeteiligung demnach keine reine Präferenzentscheidung des Verkäufers ist, sondern auch durch die Eigenschaften des zu verkaufenden Objektes beeinflusst wird, was bedeutet, dass unterschiedliche Marktsegmente unterschiedlich behandelt werden müssen. In beiden Untersuchungsgebieten werden städtische Immobilien sowie Mehrfamilienhäuser häufiger von Maklern angeboten, während die Effekte des Preises und bei Reihenhäusern in Deutschland und in Kalifornien gegenläufig sind. Bemerkenswert ist insbesondere der Unterschied, dass in Deutschland die Maklerquote bei extrem hohen oder niedrigen Preisen am größten ist, während in Kalifornien im mittleren Preissegment am häufigsten auf Makler zurückgegriffen wird.

Spannend zu beobachten wird in diesem Zusammenhang die Entwicklung der Maklerquote in den kommenden Jahren sein. Die während des Erhebungszeitraums bereits präsente COVID-19-Pandemie könnte verzerrende Effekte hervorgerufen haben. Durch die Ende 2020 und damit nach der Erhebung der hier verwendeten Daten veranlasste Einführung des Bestellerprinzips beim maklerbetreuten Verkauf von Wohneigentum wird durch die nun beidseitig anfallenden Kosten möglicherweise ein Umdenken vieler Verkäufer bei der Beauftragung eines Maklers eintreten. Einer ersten Untersuchung von Sagner und Voigtländer (2021) zufolge zeichnet sich auf Angebotsebene in der kurzen Frist bereits ein steigender Anteil provisionsfreier Angebote ab, ein Trend, der auch von Sprengnetter (2021) in großen deutschen Städten für Eigentumswohnungen bestätigt wird. Jedoch gilt es weiterhin, deutschlandweit verlässliche Daten über die Maklerquote auf Basis von tatsächlichen Transaktionen statt Angeboten zu generieren. Dies könnte über die lokalen Gutachterausschüsse und ihre Kaufpreissammlungen erreicht werden, sofern diese dem Hamburger Vorbild, wo bereits Maklerquoten im Immobilienmarktbericht kommuniziert werden (Gutachterausschuss für Grundstückswerte in Hamburg 2020), folgen würden. Solchen ergänzenden Untersuchungen sollte verstärkt Beachtung zukommen, da nur über die zusätzliche Erhebung der Maklerquote basierend auf Transaktionsdaten, der Abgleich mit Angebotsdaten möglich ist. Mit darauf basierender, zunehmender quantifizierbarer Aussagefähigkeit könnte die Forschung zur Maklerquote bis hin zu Prognosemodellen zur Beteiligung eines Maklers für individuelle Inserate ausgebaut werden.

Zusammenfassend lässt sich sagen, dass sich der Immobilienhandel in Kalifornien und Deutschland, sowohl durch die Unterschiede im Transaktionsprozess als auch durch die verschieden interpretierte Rolle des Maklers, merklich unterscheidet. Insbesondere die Betrachtung des deutschen Maklers als Interessenvertreter des Verkäufers im Gegensatz zu der doppelten Maklerbetreuung in den USA, wo er als Ersatz für den Notar dient, löst sehr unterschiedliche Wahrnehmungen in der Bevölkerung aus. Damit lassen sich Unterschiede in der Maklerquote zwischen den Ländern erklären. Dass generell viele Immobilieneigentümer auf Makler zurückgreifen, liegt nicht zuletzt daran, dass viele sich nicht persönlich um den Verkauf kümmern wollen oder können. Ob dies aus Zeitmangel, fehlendem Sachverstand oder schlicht Bequemlichkeit geschieht bleibt offen. Fakt ist, dass der Verkauf einer Immobilie in Deutschland durch die professionelle Unterstützung des Notars bei der Transaktion sowie Onlineimmobilienportalen bei der Vermarktung erheblich einfacher ist als in den USA.

## Anhang

### Herleitung Odds-Ratio für die Variable Preis pro Quadratmeter

Mit der systematischen Komponente

$$\mathrm{z}\left(\mathrm{x}\right)=\beta _{0}+\beta _{1}x_{1}+\ldots +\beta _{k}x_{k}{,}$$

ergibt sich nach Backhaus et al. (2018) die logistische Regressionsfunktion zu

$$\uppi \left(x\right)=\frac{1}{1+\mathrm{e}^{\hbox{--}({\beta _{0}}+{\beta _{1}}{x_{1}}+\ldots +{\beta _{k}}{x_{k}})}}$$

Daraus folgt für unseren Fall unter der Annahme, dass nur x_1_ und x_1_^2^ im Modell enthalten sind, für die Chancen die folgende Gleichung

$$\text{Odds}\left(x\right)=\frac{\uppi (x)}{1\hbox{--}\uppi (x)}=\mathrm{e}^{{\beta _{0}}+{\beta _{1}}{x_{1}}+{\beta _{2}}{{x_{1}}^{2}}}.$$

Betrachtet man nun eine Erhöhung von x_1_ um c Einheiten, so lauten die veränderten Chancen

$$\begin{aligned}
				\text{Odds}\left(x+c\right)&=\mathrm{e}^{{\beta _{0}}+{\beta _{1}}({x_{1}}+c)+{\beta _{2}}({{x_{1}}+c)^{2}}}\\
				\Leftrightarrow \text{Odds}\left(x+c\right)&=\mathrm{e}^{{\beta _{0}}+{\beta _{1}}{x_{1}}+{\beta _{1}}c+{\beta _{2}}{{x_{1}}^{2}}+2{\beta _{2}}{x_{1}}c+{\beta _{2}}{c^{2}}}\\
				\Leftrightarrow \text{Odds}\left(x+c\right)&=\mathrm{e}^{{(\beta _{0}}+{\beta _{1}}{x_{1}}+{\beta _{2}}{{x_{1}}^{2}})+{\beta _{1}}c+2{\beta _{2}}{x_{1}}c+{\beta _{2}}{c^{2}}}\\
				\Leftrightarrow \text{Odds}\left(x+c\right)&=\mathrm{e}^{{\beta _{0}}+{\beta _{1}}{x_{1}}+{\beta _{2}}{{x_{1}}^{2}}}e^{{c\beta _{1}}+c{\beta _{2}}\left(2x_{1}+c\right)}\\
				\Leftrightarrow \ \text{Odds}\left(x+c\right)&=\text{Odds}\left(x\right)e^{{c\beta _{1}}+c{\beta _{2}}\left(2x_{1}+c\right)}
				\end{aligned}$$

Die Odds-Ratio einer Veränderung um c Einheiten ist laut Backhaus et al. (2018) definiert als das Verhältnis der veränderten Chancen zu den vorherigen Chancen und lautet damit

$$\mathrm{OR}\left(\mathrm{c}\right)=\frac{\text{Odds}(x+c)}{\text{Odds}(x)}=\frac{\text{Odds}\left(x\right)\mathrm{e}^{{c\beta _{1}}+c{\beta _{2}}\left(2x_{1}+c\right)}}{\text{Odds}(x)}=\mathrm{e}^{{c\beta _{1}}+c{\beta _{2}}\left(2x_{1}+c\right)}.$$

Diese Formel ist dabei unverändert gültig, wenn neben *x*_*1*_ weitere erklärende Variablen hinzukommen, da diese sich als Teil von Odds(*x*) ebenfalls wegkürzen.

## Footnotes

1 Die rechtlichen Einzelheiten des Transaktionsprozesses unterscheiden sich in den einzelnen Bundesstaaten durch individuelle Gesetze nicht unerheblich. Deshalb wird der Prozess exemplarisch für den bevölkerungsreichsten Bundesstaat Kalifornien beschrieben und im Weiteren Kalifornien als abgegrenzte Untersuchungsregion innerhalb der USA gewählt.

2 Die Umrechnung erfolgte zu einem Kurs von 100 USD = 84,42 € per 11. September 2020 (https://kurse.boerse.ard.de/ard/kurse_einzelkurs_uebersicht.htn?i=2079559).

3 Auf Grund der notwendigen Umkodierung der kategorialen Variable Immobilientyp in Dummyvariablen und der damit verbundenen Auslassung einer Ausprägung ermittelt sich der Anteil der Einfamilienhäuser als efh = 1 − mfh − rh − dhh.

4 Bei einer näheren Untersuchung dieser und ähnlich teurer Objekte ist aufgefallen, dass es sich dabei nicht um Datenfehler, sondern um Luxusimmobilien in den bekanntesten Lagen des Landes handelt. Darunter etwa Malibu Beach in Kalifornien und die Insel Sylt in Deutschland.

5 Erfolgswahrscheinlichkeit: $(\mathrm{makler}_{i}=1)=\pi$; Chancen (Odds) für $x_{j}=1$: $\pi \left(x_{j}=1\right)/(1-\pi \left(x_{j}=1\right))$ Chancenverhältnis (Odds-Ratio): ($\textit{odds f{\"u}r}\ x_{j}=1)/(\textit{odds f{\"u}r}\ x_{j}=0)$

6 An dieser Stelle werden deutlich mehr Dezimalstellen angegeben als in der Übersichtstabelle, da wie bereits erklärt eine Änderung des Preises um einen Euro pro Quadratmeter eine unerhebliche Änderung auslöst. Um bei den nachfolgend betrachteten größeren Änderungen keine Rundungsungenauigkeiten fortzuführen, wird die Anzahl der Dezimalstellen erhöht.

7 Basierend auf den geschätzten Koeffizienten und den geschätzten Standardabweichungen wurde ein Welch-Test (Zwei-Stichproben-t-Test bei ungleichen Standardabweichungen) auf Gleichheit der Koeffizienten unter der Nullhypothese, dass Gleichheit vorliegt, durchgeführt. Das Ergebnis liefert *p* < 0,0001.
